# Use of cryoablation beyond the prostate

**DOI:** 10.1007/s13244-015-0460-7

**Published:** 2016-01-13

**Authors:** Saim Yılmaz, Mustafa Özdoğan, Metin Cevener, Ali Ozluk, Aysegul Kargi, Feride Kendiroglu, Irfan Ogretmen, Akin Yildiz

**Affiliations:** MIIO Group, Radiology Division, Memorial-MedStar Hospitals, Antalya, Turkey; MIIO Group, Medical Oncology Division, Memorial-MedStar Hospitals, Antalya, Turkey; MIIO Group, General Surgery Division, Memorial-MedStar Hospitals, Antalya, Turkey; MIIO Group, Nuclear Medicine Division, Memorial-MedStar Hospitals, Antalya, Turkey; MIIO: Mediterranean Integrative and Innovative Oncology, Antalya, Turkey

**Keywords:** Ablation techniques, Lung cancer, Renal cancer, Liver cancer, Cryoablation

## Abstract

**Abstract:**

Cryoablation has been used for many years as a surgical ablation technique in the prostate and kidney. However, since the introduction of high-intensity focused ultrasound (HIFU) and robotic surgery for prostate tumours, its popularity in the urologic community has declined. In the early 2000s, innovations in cryoablation technology allowed the use of thinner probes, which were suitable for percutaneous application. As a result, radiologists began using cryoablation, first in the liver, and then in other organs or tissues such as the kidney, lung, breast, pancreas, bone, and soft tissue. In most of these locations, cryoablation has great potential given its inherent advantages, including the use of local anaesthesia, little or no pain during and after the procedure, real-time monitoring of the ablation area on US, CT or MRI, the potential for ablation of large tumours with multiple probes, and the ability to change the shape of the ablation in non-spherical tumours. Yet despite these advantages, the use of percutaneous cryoablation among radiologists appears to be far lower than that of heat-based ablation techniques. The aim of this article is to outline specific aspects of cryoablation and to illustrate its potential clinical applications with case presentations.

***Key Points*:**

• *Recent advances have made cryoablation suitable for percutaneous use by radiologists with image guidance.*

• *Cryoablation has distinct advantages over heat-based ablation techniques.*

• *Cryoablation is becoming increasingly popular for lung, breast, kidney, bone, and soft tissue tumours.*

## Introduction

Cryoablation is a well-known ablation technique which uses rapid freezing and thawing sequences for tissue destruction. In the current third-generation devices, this is performed by rapid decompression of argon gas (Joule–Thomson effect) and thawing, either passively or by circulation of helium gas through the probe. These freezing and thawing cycles cause intracellular and extracellular ice crystal formation, intracellular dehydration, and ischemia due to vascular thrombosis, all of which may result in necrosis or apoptosis [1, 2].

Percutaneous cryoablation is typically performed under mild sedation and local anaesthesia. Cryoprobes are normally inserted into the lesion under ultrasound (US) or computed tomography (CT) guidance.

**Fig. 1:**
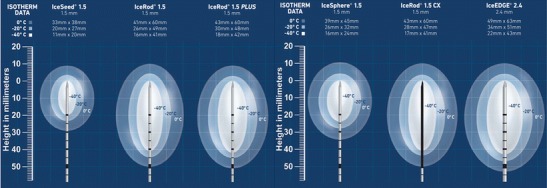
Some of the commercially available cryoprobes and the size, shape, and thermal aspects of their iceballs (Courtesy of Galil Medical)

The number of probes depends on the size of the lesion, and multiple probes are frequently necessary for the best coverage. In a typical cryoablation procedure, a 10-min freezing, 5–10-min thawing, and 10-min freezing protocol is performed, although other protocols have been used in some organs. During freezing, an iceball is formed around the probes, which is generally visible on US, CT, and magnetic resonance imaging (MRI) in all tissues except healthy bone. The temperature at the outer surface of the iceball is 0 °C, with cell death reliably occurring at a depth of 3–5 mm near the edge. During the freezing process, the size and shape of the iceball can be monitored to ensure that it covers the lesion completely and is not near any critical structures. When necessary, these structures can be protected by fluid, air, or balloon interposition, or by reducing the power of the nearest probe. During ablation, iceball formation can be monitored with US, CT, or MRI. US provides real-time monitoring, but only the superficial border of the iceball is visible. CT and MRI provide excellent visualization of the iceball, but require repeated scanning during the procedure. In general, US and CT are preferred for the guidance and monitoring of cryoablation, since MRI is less practical in light of the need for special equipment and the limitations around the magnetic field. US is usually sufficient for superficial lesions located in noncritical areas such as the breast, thoracic and abdominal wall, and liver, while CT is preferred in the lung, kidney, bone, and deep visceral organs. In many clinical scenarios, the combined use of US and CT may be the best solution for guiding probe placement and iceball monitoring. After ablation is completed, the probes are actively thawed with helium and then removed. With some of the latest-generation probes, it is also possible to perform track ablation at temperatures over 220 °C [1–4] (Fig. 1).

Cryoablation offers certain unique advantages over the other ablation techniques. First, because of the natural anaesthetic effect of the cold, the procedure can be performed under local anaesthesia. This is important in cases where general anaesthesia or deep sedation are dangerous for the patient, breath-holding is desired during the procedure, or ablation is performed in an office setting. In addition, patients experience much less pain after treatment with cryoablation than with radiofrequency. Second, in contrast to heat-based ablation methods such as radiofrequency, microwave, or laser, tumours larger than 3–5 cm in size can be reliably ablated with the use of multiple probes (up to 25 probes can be used simultaneously). Third, the iceball is readily visible on CT, MRI, and US, which allows real-time monitoring of the ablation area. With clear demonstration of the treatment margin, tumours close to critical structures such as nerves, bowel, or bladder may be treated confidently and with greater safety. And forth, cryoablation causes less damage to collagen fibres, which makes it an attractive choice in tumours near critical structures [1–4].

However, cryoablation has two major disadvantages. First, it is more expensive than other ablation methods, especially if multiple probes are used. The use of argon and helium is another factor that increases cost. And second, it is more time-consuming than the other methods, which is particularly important if multiple ablations are performed in the same session [1].

In the past, cryoablation was used almost exclusively by surgeons, particularly urologists, mainly because the commonest indication was prostate cancer and the probes were large, required liquid nitrogen for cooling, and were not well insulated, rendering them unsuitable for percutaneous application [1, 5]. In the early 2000s, the introduction of thinner argon-based probes with insulation along their shafts ushered in the era of image-guided percutaneous cryoablation. Radiologists first tested cryoablation in the liver; however, due to the presence of already established methods such as radiofrequency and associated complications such as cryoshock, it was not widely used in this organ [2]. For the last decade, the growing indication of percutaneous therapies in oncology has enabled the use of cryoablation in other organs and tissues, including the lung, kidney, breast, pancreas, soft tissue, and bone [1–4, 6]. Although heat-based techniques like radiofrequency and microwave have been more commonly used in these locations as well, cryoablation is increasingly preferred given its unique features mentioned earlier. Yet there is little data in the literature on the use of image-guided percutaneous cryoablation by radiologists. This article reviews the indications and results of cryoablation in locations other than the prostate, and highlights its advantages and disadvantages compared to other ablation techniques.

## Cryoablation in lung tumours

In early non-small cell lung cancer (NSCLC) and lung oligometastases, the classic ablation technique is radiofrequency (RF) [7]. It is well-known, however, that due to the high electrical resistance and poor thermal conductivity of alveolar air, RF is less successful in the lung than in the liver. Microwave ablation, another heat-based ablative technique, has been proposed to overcome the limitations of RF. However, data on microwave ablation in the literature are sparse, and high complication rates have been reported [7, 8]. Cryoablation has been successfully used within the last decade in primary and secondary lung tumours, with 2-year local progression-free survival rates of 60.7–100 % in early NSCLC and 45.6–80.4 % in metastases [9, 10]. In the lung, a triple-freeze protocol is generally preferred to the classic dual-freeze protocol, as the former was shown in an animal study to be associated with faster and larger ablation [10].

**Fig. 2:**
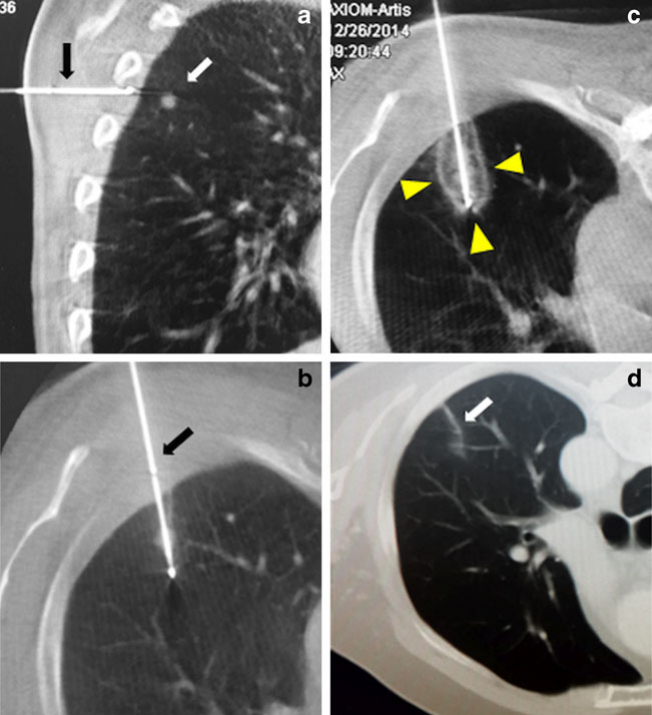
Cryoablation of a lung metastasis. **a** The sagittal cone beam CT image shows a 7-mm biopsy-proven colon cancer metastasis (*white arrow*) and the cryoablation needle (*black arrow*). **b** Under local anaesthesia, the cryoablation needle is percutaneously inserted into the lesion (*black arrow*). **c** During ablation, an iceball (*arrowheads*) is formed that covers the lesion. **d** After 1 year, the control CT image shows a fibrotic remnant (*white arrow*) in place of the tumour

The advantages of cryoablation in the lung include the ability to perform the ablation under local anaesthesia with breath-holding, which is important for successful ablation of small lesions located near the diaphragm, as well as the visibility of the ablation area (iceball) on CT and little or no pain during or after the procedure [7–10] (Fig. 2). The absence of major damage to collagen fibres makes cryoablation an attractive option for lesions located near the mediastinum, pericardium, diaphragm, or pleura. In tumours that have infiltrated the chest wall, cryoablation is superior to other ablation methods in that it causes less pain and little damage to the connective tissue [9].

Common complications of lung cryoablation are similar to those of RF and microwave, and include pneumothorax, infection, and pleural fluid. However, haemoptysis during or after the procedure is more common in cryoablation [9], and is more likely to occur in central lesions and when multiple cryoprobes are used. Since haemoptysis may last for days or even weeks after ablation, the use of antiaggregants or anticoagulants is not appropriate during this period. Therefore, cryoablation may not be a good choice in patients who depend on such medications.

## Cryoablation in the breast

**Fig. 3:**
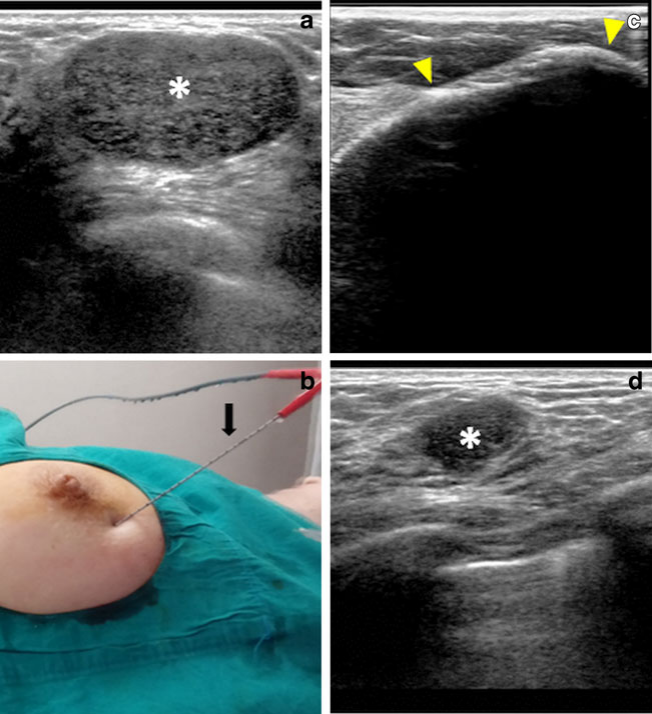
Cryoablation of a fibroadenoma. **a** The US image shows a 31×21×16-mm fibroadenoma (*) in the left breast of a 33-year-old female. **b** The cryoablation needle (*black arrow*) is inserted percutaneously into the lesion. **c** During ablation, the US image shows the typical iceball (*arrowheads*) encompassing the lesion. The skin is protected by external warming and subcutaneous fluid injection. **d** Seven months later, the follow-up US shows marked shrinkage of the fibroadenoma (*)

The classic indication of cryoablation in the breast is fibroadenoma. These benign tumours are quite common in young women and do not require treatment in most cases. In routine practice, however, surgery may be performed for fibroadenomas that are large, symptomatic, or with atypical US features. Since it may cause scarring and deformation, surgery is not desirable for young women, particularly in the case of multiple fibroadenomas [11]. Cryoablation, which was approved by the U.S. Food and Drug Administration (FDA) in 2002 for the treatment of fibroadenomas, is an excellent alternative in such cases. The procedure is painless, performed under local anaesthesia, and able to treat multiple fibroadenomas in the same session. Research has shown that more than 80 % of fibroadenomas become non-palpable 1 year after cryoablation [12] (Fig. 3).

**Fig. 4:**
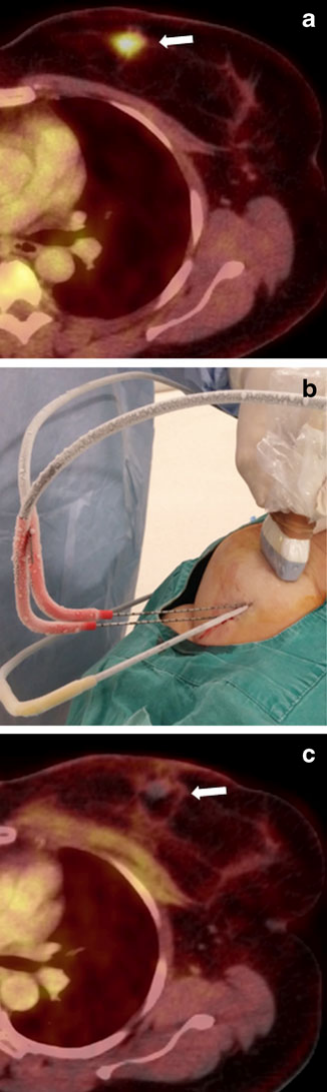
Cryoablation of breast cancer. **a** The PET-CT image shows a 2-cm FDG-avid lesion (*arrow*) in the left breast, proven to be invasive ductal carcinoma. The patient also has a 3-cm lytic metastasis in the right distal femur (not shown). **b** Under local anaesthesia and US guidance, cryoablation of the breast mass is performed using three cryoneedles. **c** Two months later, the follow-up PET-CT shows complete metabolic response in the lesion (*arrow*). The ablation area was then percutaneously removed with vacuum biopsy, which revealed fibrosis and fat necrosis

An emerging indication of cryoablation is breast cancer. In the last decade, percutaneous US-guided ablation has been used successfully in the treatment of breast cancer [13–15]. Ideal patients for ablation are elderly women with small (<2 cm) tumours and no axillary involvement or distant metastasis. In patients with oligometastases, percutaneous ablation is no longer considered contraindicated, provided that they are also manageable percutaneously [13]. The most popular ablation methods in breast cancer are RF, cryoablation, and high-intensity focused ultrasound (HIFU). Although HIFU is completely non-invasive, it is not suitable for lesions close to the skin or pectoralis muscle, requires expensive equipment, and is associated with pain and long ablation times, frequently requiring deep sedation or general anaesthesia to keep the patient compliant. Therefore, RF and cryoablation are more commonly used. Although their success rates are similar, cryoablation has certain advantages. First, it can be performed under local anaesthesia, which is important for older patients. Second, the ablation area can be enlarged with the use of multiple (generally 2–3) probes, which may decrease the rate of local recurrence (Fig. 4). Third, the visibility of the superficial border of the iceball on US enables easier and more reliable skin protection. And fourth, because it causes less damage to collagen tissue, pectoralis muscles are generally unaffected during the ablation of deep lesions [11–15]. A recent review including seven studies reported short-term local control in 73 % of patients, with better results in small (<15 mm) ductal tumours and when multiple cryoprobes were used [16].

## Cryoablation in bone and soft tissue tumours

**Fig. 5:**
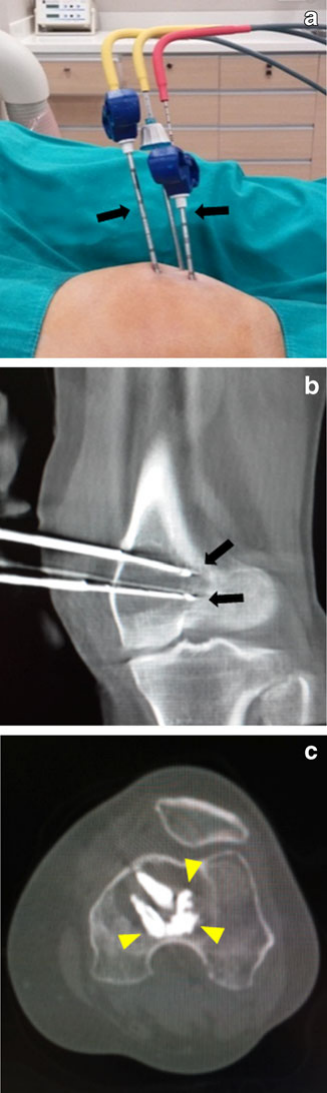
Cryoablation of bone metastasis (the solitary metastasis in the distal femur of the previous patient). **a**, **b** First, under local anaesthesia and US guidance, three bone biopsy needles are inserted into the lesion under cone-beam CT guidance, and cryoablation is then performed with needles (*arrows*) inserted coaxially. **c** After the ablation, the cryoneedles are removed, and cement (*arrowheads*) injection is performed through the outer needles to support the bone

RF is the most common ablation method in bone and soft tissue tumours [1, 17], and in recent years has become the gold standard in the treatment of osteoid osteoma [17]. Cryoablation is also becoming increasingly popular for percutaneous ablation of bone and soft tissue tumours. Although both cryoablation and heat-based ablation techniques provide safe, effective, and durable results, advantages such as real-time visibility of the ablation area, the ability to treat large tumours with multiple probes, and the absence of significant pain during and after the procedure render cryoablation a more attractive alternative for bone and soft tissue lesions [17–19] (Fig. 5).

Like any other ablation method, cryoablation must be used with caution for lesions near the major nerves, especially in the upper thoracic, neck, and pelvic regions. When necessary, nerve protection techniques such as fluid, air, or carbon dioxide instillation, balloon interposition, or cryoprobe retraction/torquing should be applied to prevent nerve injury. Because of its superior visualization of the ablation zone, cryoablation is superior to other methods for ablation of a tumour near a critical nerve [17, 18].

Percutaneous cryoablation has been successfully used in the palliation of painful bone metastases [19]. Although radiotherapy is the standard treatment in these patients, it provides pain relief in only about 60 % of cases, requires several weeks to occur, and is often temporary. HIFU is an approved alternative which, like radiotherapy, is non-invasive. Because ultrasound energy is highly absorbed by the bone, the periosteal nerves are easily destroyed, resulting in rapid pain relief. However, HIFU cannot ablate the tumour under an intact periosteum, and therefore is less effective in deeply seated bone tumours. In contrast, cryoablation can destroy both the periosteum and the tumour, which may allow palliative as well as curative treatment, and will likely produce more durable pain reduction. In weight-bearing bones in the spine and pelvis, the combined use of cementoplasty and cryoablation may provide additional pain relief and reduce the risk of pathologic fracture [17, 19].

Cryoablation has recently been reported as an alternative local treatment for desmoid tumours [17, 18]. Although complete ablation was not always possible, cryoablation was proven safe and effective for the local control of these difficult tumours [18]. There are also reports in the literature of the use of cryoablation in the treatment of aneurysmal bone cysts, giant cell tumours, osteoid osteoma, non-ossifying fibroma, and soft tissue vascular malformations [1, 16, 17]. Another potential area for cryoablation is percutaneous neurolysis in facet joint syndrome and degenerative spinal pain, although RF is more commonly used for these indications [20].

**Fig. 6:**
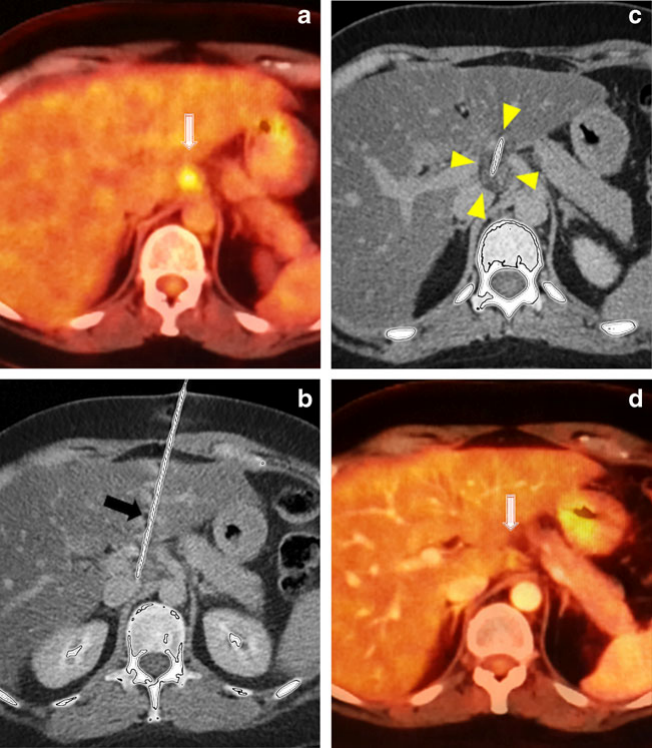
Cryoablation of a peripancreatic lymph node. **a** In a 44-year-old patient after surgical treatment of ovarian carcinoma, PET-CT image shows a lymph node metastasis (*white arrow*) near the pancreas. Since the metastasis was solitary, percutaneous ablation was planned, and because of the critical location of the lesion, cryoablation was preferred. **b** Under local anaesthesia and US+CT guidance, a cryoablation needle (*black arrow*) was inserted transhepatically into the lesion. **c** The lymph node was ablated with two freeze and one thaw cycle, and an iceball was typically seen (*arrowheads*). Since the liver was traversed, track ablation was performed after cryoablation to prevent bleeding and tumour seeding. **d** Eight months later, the follow-up CT shows complete metabolic response of the lesion (*white arrow*)

Cryoablation has also been reported for the local control of inguinal lymph node metastases [21]. Although RF is the classic ablation method in lymph nodes, cryoablation may be an attractive option in critical locations (Fig. 6).

## Cryoablation in renal tumours

With the widespread use of cross-sectional imaging such as CT and MRI, incidental small (<4 cm) renal tumours are increasingly detected. Since these tumours are diagnosed at an early stage (generally 1A) and generally in the elderly, percutaneous ablation is a suitable option. In the last decade, both RF and cryoablation have been used extensively to treat small renal cell carcinoma (RCC). The classic indications for renal ablation are comorbid conditions preventing surgery, contralateral recurrence, and hereditary precancerous conditions. The long-term (5–10 years) results with these techniques have shown an oncologic outcome equal to that of surgery, with fewer complications and less decline in renal function [22–25].

**Fig. 7:**
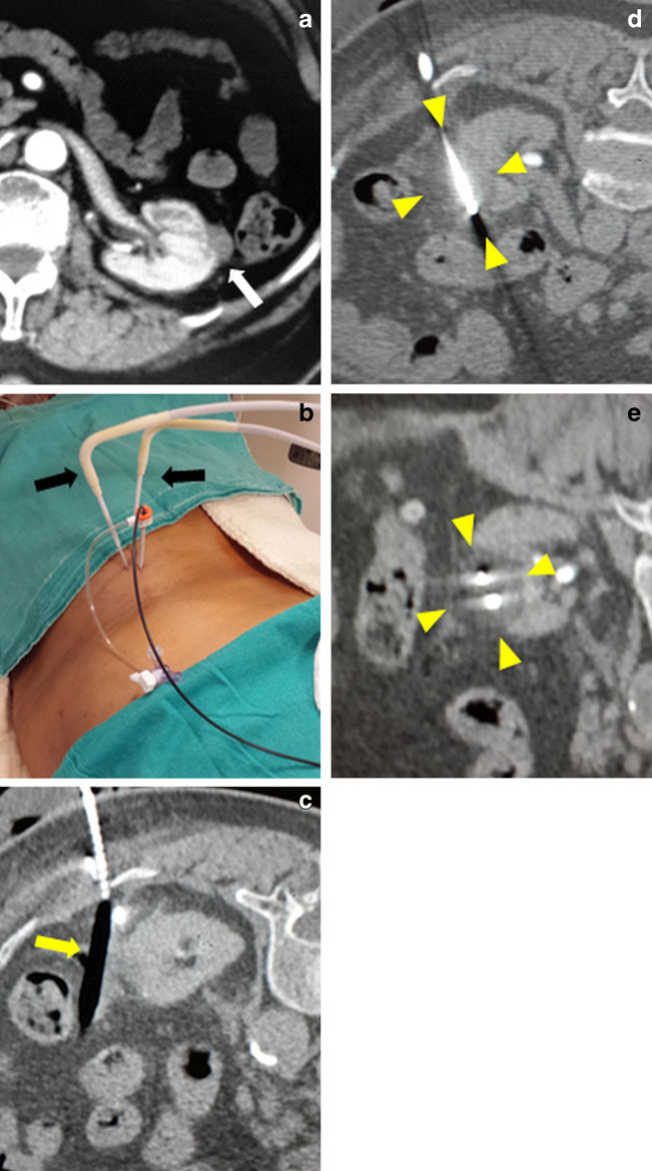
Cryoablation of recurrent renal cancer. **a** The axial contrast-enhanced CT image shows an exophytic renal tumour (*white arrow*) next to the descending colon. The patient had undergone ipsilateral partial nephrectomy for RCC 6 months earlier. **b**, **c** Under local anaesthesia and US+BT guidance, two cryoneedles are inserted percutaneously (*black arrows*) into the lesion. The tumour is separated from the descending colon with an angioplasty balloon (*yellow arrow*) inserted percutaneously through a sheath. **d**, **e** Axial and reformatted CT images show the iceball (*arrowheads*) covering the tumour and the two cryoablation needles. During the 1.5-year follow-up, there was no evidence of tumour recurrence

In the past, renal cryoablation was a “surgical” procedure, typically performed laparoscopically by urologists [22]. With the introduction of argon-based thin probes in the 2000s, radiologists—who were already performing image-guided RF ablation for renal tumours—also began performing percutaneous renal cryoablation [24, 25]. Studies comparing laparoscopic and percutaneous cryoablation have shown oncologic results that were similar, but the latter had lower rates of complications. Studies comparing RF ablation with cryoablation found similar results in terms of oncologic outcome and complication rates. The types of complications, however, differed slightly between these two techniques: chyluria was more common with RF, and bleeding was more common with cryoablation, both of which were treated conservatively [23–25]. A unique advantage of cryoablation is the visibility of the ablation area (iceball) and the potential for safe displacement of tissue using air-filled balloons, which are not harmed by the ice (Fig. 7).

## Cryoablation in adrenal tumours

Primary adrenal tumours such as functioning adenoma and carcinomas are normally treated with laparoscopic adrenalectomy, with percutaneous ablation limited to cases unsuitable for surgery [26]. In adrenal metastases (isolated or oligometastatic), percutaneous image-guided ablation has a well-established role, and has been performed using RF, microwave, and cryoablation with similar success [26–28].

**Fig. 8:**
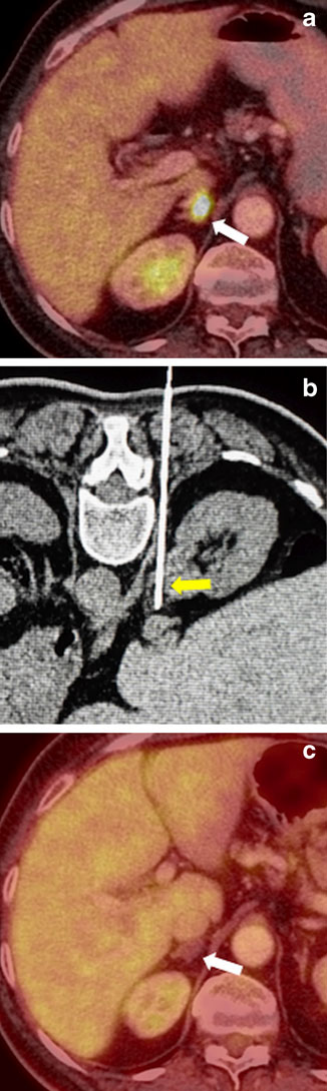
Cryoablation of a surrenal gland metastasis. **a** The PET-CT image shows an FDG-avid lesion (*white arrow*) in the right surrenal gland. The patient had undergone surgery for rectal cancer 1 year earlier. **b** During the cryoablation, the tilted axial image shows the ablation needle (*yellow arrow*) directed towards the lesion (*arrow*). **c** One year after the cryoablation, the follow-up PET-CT shows no evidence of tumour

The procedure is typically performed under CT guidance with the patient in prone or lateral decubitus position (Fig. 8). One specific complication of adrenal ablation is hypertensive crisis, for which pre- and peri-procedural alpha and beta blockers are recommended [26, 27]. In RF and microwave procedures, the hypertensive crisis occurs during the ablation, and is more pronounced with microwave, since the tissue is heated more rapidly. In cryoablation, the hypertensive crisis is generally less aggressive and typically occurs during thawing [27, 28].

## Cryoablation in liver tumours

**Fig. 9:**
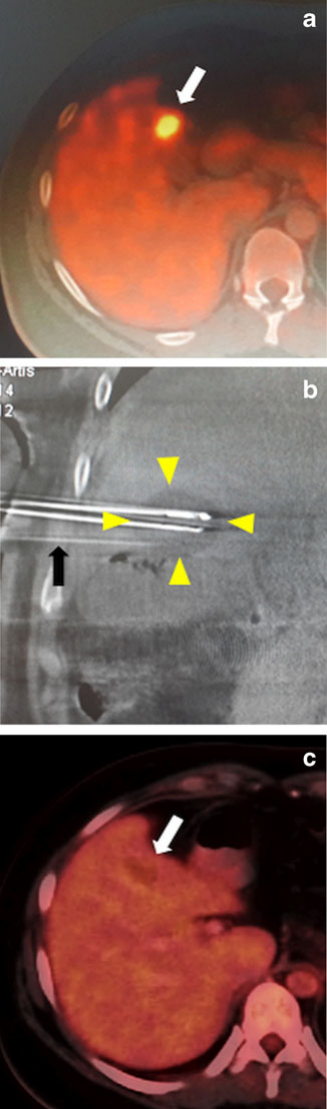
Cryoablation in the liver. **a** The axial PET-CT image shows a subcapsular FDG-avid lesion (*white arrow*). The patient had previously undergone surgery for cholangiocarcinoma, and the biopsy showed the lesion to be metastatic cholangiocarcinoma. **b** Cryoablation was performed under US and cone-beam CT guidance, which shows the iceball (*arrowheads*) produced by two cryoneedles covering the lesion. Another needle (*black arrow*) was also inserted for fluid injection to separate the adjacent transverse colon from the tumour. **c** One year later, the control PET-CT image shows complete metabolic response in place of the lesion

Cryoablation has been extensively used in liver tumours, particularly in difficult cases such as those located in subcapsular, subdiaphragmatic, or central regions [29] (Fig. 9). Although successful results have been reported in both hepatocellular carcinoma (HCC) and colorectal metastases [29, 30], several factors limit its use in the liver. First, cryoablation can cause what is referred to as the cryoshock phenomenon, which is a cytokine-mediated systemic reaction characterized by hypotension, disseminated intravascular coagulopathy, and multiorgan failure. Cryoshock is generally seen after cryoablation of large (>5 cm) hepatic tumours and is frequently fatal. Second, since the liver is a well-perfused organ, there is a significant “cold sink” effect, which results in a smaller iceball. Thus, a greater number of needles are required to obtain a certain ablation size, which may increase both cost and risk of complications. And third, heat-based ablations such as RF and microwave are highly successful in the liver, and are also safer and cheaper. Therefore, these systems are currently considered the gold standard in primary and metastatic liver tumours, and cryoablation is reserved for select cases [29–31].

## Cryoablation in other locations

Cryoablation has also been reported in the literature for pancreatic cancer [32, 33]. Although RF has been more commonly used, and irreversible electroporation has recently become popular in pancreatic cancer, both techniques have been associated with significant risk of complications [34, 35]. Pancreatic cryoablation, on the other hand, was found to be safer, with delayed gastric emptying the only reported complication [32].

In general, virtually any solid tumour in a location suitable for percutaneous ablation can be treated with cryoablation. Hypervascular tumours, however, are likely not good candidates for cryoablation, as iceball formation is limited due to the cold sink effect, and the use of multiple probes carries a greater risk of vascular complications such as bleeding and arteriovenous fistula. In such cases, heat-based ablation may be more suitable, as the vessels are occluded during heating and track ablation.

## Conclusion

After a long history of urologic use and a decline in popularity, cryoablation is back again as an image-guided percutaneous technique, thus providing an opportunity for radiologists. In this article, we tried to summarize the classic and emerging indications of percutaneous cryoablation in various organs, and to emphasize its advantages and limitations in comparison to heat-based ablation. In many tumours, cryoablation may be preferable to other ablation methods, given advantages such as the use of local anaesthesia, less pain during and after the procedure, visibility of the ablation area on US, CT, and MRI, the potential to ablate large tumours with multiple probes, and the ability to change the shape of the ablation in non-spherical tumours. As pioneers in image-guided minimally invasive treatments, radiologists should take advantage of these unique features of cryoablation for the percutaneous treatment of tumours.
